# Permanganate of Potash in Gonorrhœa

**Published:** 1865-07

**Authors:** 


### PERMANGANATE OF POTASH IN GONORRHŒA.

Dr. J. G. Rich, of Beachville, Canada West, states (*Canada Lancet*, July 15, 1864,) that he has frequently employed, during the past two years, the permanganate of potash as an injection for gonorrhœa, and with the most satisfactory results, in some cases having effected a cure in forty-eight hours.

His usual mode of treatment is as follows: "℞—Potassæ bitart. ℈j; Podophyllin, gr. j.—M. In chart. No. IV. divid. S. One every two hours until free catharsis is produced.

"After which, ℞—Potassæ permangan. gr. vj; Aquæ fontan. ℥j.—M. S. To be used as an injection three times a day.

"I direct at the same time the free employment of mucilaginous drinks, as althæa, ulmus, acacia, etc., and put the patient upon a non-stimulating regimen.

"Out of sixty-four registered cases this treatment has failed in but two instances. And I find that recent attacks usually become arrested by it after from three to six injections. I have found it advisable to continue the demulcents for at least a week after the cessation of the discharge. In none of all these cases was the injection continued after the fourth day.

"When accompanied by chordee, I usually employ the following: ℞—Lupulin, ℈j; Micæ panis, q. s.—M. Ft. mass. in pilulus xvi, dividenda. S. Two, three, or four on going to bed."

### PERSULPHATE OF IRON IN HEMORRHOIDS.

Dr. Geo. S. Cartwright, Asst. Surg. U. S. V., highly extols (*Cincinnati Lancet and Observer*, May, 1864,) the efficacy of the persulphate of iron employed as an ointment in the treatment of hemorrhoids. It is especially beneficial, he states, in ulcerated hemorrhoids; or in those whose constitutions are debilitated from diarrhœa, long marches, and excessive fatigue of any kind.

Of several cases which he relates illustrative of the advantages of this remedy, we select the following:

"Major ——, U. S. A., of full habit, has been the subject of slight hemorrhoids for several years. For the last twelve months, has been obliged to travel a greater part of the time in a rough vehicle. Applied to me December 5th, 1863. On examination, found a small tumor, external to the sphincter, about the size of a large pea; when at stool it would protrude to the size of a small walnut, and would with difficulty be returned.

"*Treatment.*—Lead water freely applied to the part, and ℞ ferri persulphas ʒ ss., cerate simplex ℥ j. Rub well together and apply on retiring at night. The effect of the persulphas was almost immediate, relieving pain and cauterizing the part.

"I would state that he had previously used ointment of galls, tannin, opium, etc., with only a temporary relief. The effect of the persulphas is permanent, and in the above case he was able to ride on horseback, or take active exercise, within two weeks after commencing the use of the iron, without the least inconvenience. It is now two months since he first commenced the use of it and has not had any return since."

Dr. C. sometimes employs the ointment with double the proportion of the persulphate used in this case.

